# Anti-malarial ozonides OZ439 and OZ609 tested at clinically relevant compound exposure parameters in a novel ring-stage survival assay

**DOI:** 10.1186/s12936-019-3056-8

**Published:** 2019-12-18

**Authors:** Annabelle Walz, Didier Leroy, Nicole Andenmatten, Pascal Mäser, Sergio Wittlin

**Affiliations:** 1 Swiss Tropical and Public Health Institute, Socinstrasse 57, 4002 Basel, Switzerland; 2 University of Basel, Petersplatz 1, 4001 Basel, Switzerland; 3 Medicines for Malaria Venture, Route de Pré Bois 20, 1215 Geneva, Switzerland

**Keywords:** Malaria, RSA, *Plasmodium falciparum*, *kelch 13*, Cam3.I^R539T^, Artemisinins, Ozonides

## Abstract

**Background:**

Drug efficacy against *kelch 13* mutant malaria parasites can be determined in vitro with the ring-stage survival assay (RSA). The conventional assay protocol reflects the exposure profile of dihydroartemisinin.

**Methods:**

Taking into account that other anti-malarial peroxides, such as the synthetic ozonides OZ439 (artefenomel) and OZ609, have different pharmacokinetics, the RSA was adjusted to the concentration–time profile of these ozonides in humans and a novel, semi-automated readout was introduced.

**Results:**

When tested at clinically relevant parameters, it was shown that OZ439 and OZ609 are active against the *Plasmodium falciparum* clinical isolate Cam3.I^R539T^.

**Conclusion:**

If the in vitro RSA does indeed predict the potency of compounds against parasites with increased tolerance to artemisinin and its derivatives, then the herein presented data suggest that following drug-pulses of at least 48 h, OZ439 and OZ609 will be highly potent against *kelch 13* mutant isolates, such as *P. falciparum* Cam3.I^R539T^.

## Background

Reduced in vivo susceptibility of *Plasmodium falciparum* to semisynthetic artemisinin derivatives was first reported a decade ago in Cambodia, where patients displayed prolonged parasite clearance rates following artesunate treatment [1, 2]. Since then, many more reports have confirmed those in vivo findings [3], and the prolonged clearance rates were found to be associated with point mutations in the *P. falciparum kelch 13* gene [4, 5]. Activity against *kelch 13* mutants is, therefore, a prerequisite for a new anti-malarial drug candidate. However, initially there was no in vitro susceptibility test that would pick up the increased tolerance to artemisinin derivatives [6]. This gap has been closed by Witkowski et al. with the development of the so-called ring-stage survival assay (RSA) [7]. In the standard RSA, 0–3 h post-invasion *P. falciparum* ring-stage parasites are incubated for 6 h at a dihydroartemisinin (DHA) concentration of 700 nM—conditions considered pharmacologically relevant.

Taking into account that other anti-malarial peroxides, such as the synthetic ozonide OZ439 (artefenomel), have different pharmacokinetic properties than the artemisinins [8], the aim of this study was to perform RSAs under clinically relevant conditions based on published concentration–time profiles of DHA and ozonides after single-dose treatment in clinical trials. The authors are aware that the single-dose scenario is not reflecting the standard anti-malarial treatment, where patients receive multiple dosings of artemisinins and in combination with a partner compound. The presented data should, therefore, be interpreted accordingly.

## Methods

### Parasite cultivation

The artemisinin-resistant *P. falciparum* isolate Cam3.I^R539T^ from Battambang, Cambodia was obtained from BEI Resources with the accession number MRA-1240. The corresponding revertant clone Cam3.I^rev^ was kindly provided by David Fidock. Parasites were cultivated in standard culture medium, consisting of hypoxanthine (50 mg/l), RPMI (10.44 g/l) supplemented with HEPES (5.94 g/l), Albumax (5 g/l), sodium bicarbonate (2.1 g/l) and neomycin (100 mg/l) [9].

### [^3^H]hypoxanthine incorporation assay

The in vitro anti-malarial activities of compounds were measured using the modified [^3^H]hypoxanthine incorporation assay [9]. Compounds were dissolved by sonication in DMSO (10 mg/ml) and diluted in hypoxanthine-free culture medium. Infected erythrocytes (100 μl per well with 2.5% haematocrit and 0.3% parasitaemia) were added to each drug titrated in 100 μl duplicates over a 64-fold range. After 48 h incubation, 0.25 microCi of [^3^H]hypoxanthine in 50 μl culture medium was added and plates were incubated for an additional 24 h. Parasites were harvested onto glass-fiber filters and radioactivity was counted using a Betaplate liquid scintillation counter (Wallac, Zurich). The results were recorded as counts per minute (cpm) per well at each drug concentration and expressed as a percentage of the untreated controls. Fifty and ninety percent inhibitory concentrations (IC50 and IC90) were estimated by linear interpolation [10].

### Ring-stage survival assay

Using the ring-stage survival assay (RSA) adapted from Witkowski et al. [7], drug potency was measured against the Cambodian *P. falciparum* clinical isolate Cam3.I, which carries the *kelch 13*-propeller mutation R539T (Cam3.I^R539T^) and the corresponding revertant Cam3.I^rev^. Cam3.I^rev^ is a Cambodian field isolate, in which the *kelch 13* mutation R539T was replaced with the reverted wild-type allele [5]. In brief, parasites were synchronized to 0–3 h post-invasion rings using 5% d-Sorbitol and a 75% Percoll cushion.

In the case of the conventional microscopic readout, 0–3 h post-invasion rings (final haematocrit 1.8% and parasitaemia between 0.5 and 1%) were exposed to a drug pulse of 6 h in standard culture medium in 48-well plates (Corning, Cat. No. 3548). Then, the content of each well was transferred to a 1.5 ml Eppendorf tube and residual compound was removed by washing four times in 1 ml of standard culture medium, before the parasites were cultivated for another 66 h in the same medium in a new 48-well plate. Afterwards, a thin blood smear was made from each well, parasitaemia was determined microscopically by counting the number of parasitized cells in approximately 10,000 cells, and the survival rate was calculated as percentage of the untreated controls.

In the case of the [^3^H]hypoxanthine incorporation method, 0–3 h post-invasion rings (final haematocrit of 1.6% and parasitaemia between 0.1 and 1.2%) were exposed to a drug pulse of 6, 20 or 48 h in standard culture medium in 96-well plates (Sarstedt, Cat. No. 83.3924). The content of all wells was then transferred to deep-well plates (Sigma-Aldrich, Cat. No. P8241-50EA) and compounds were removed through extensive washing with hypoxanthine-free culture medium (6 × 1 ml). The complete removal of compound after washing was verified by incubating the supernatant recovered after the last washing step with fresh cultures of NF54 (airport strain from The Netherlands, kindly provided by F. Hoffmann-La Roche Ltd) parasites for 72 h, ensuring that no growth inhibition was detected. After washing, drug-free parasites were re-incubated in hypoxanthine-free medium for 22 h. Then, 0.5 microCi of [^3^H]hypoxanthine in 50 μl of culture medium was added. Twenty-four hours after this [^3^H]hypoxanthine incorporation period, the assay was terminated by freezing the plates at − 20 °C. The thawed plates were harvested using a FilterMate Harvester (Perkin Elmer, Waltham, USA) and counts per minute (cpm) were obtained for each well by using the MicroBeta2^®^ 2450 Microplate Counter (Perkin Elmer, Waltham, USA). For each drug and concentration, the survival rate was calculated as percentage of the untreated controls. Three individual experiments were performed in technical duplicates.

## Results

The minimal inhibitory concentration (MIC, defined as the concentration at which the parasite multiplication factor per asexual cycle equals one) is a critical concentration determinant. Here, the in vitro 90% inhibitory concentration (IC90) was used as proxy of the MIC, as recently proposed [11]. The inhibition curves of DHA, OZ277, OZ439 and OZ609 had previously been determined by the [^3^H]hypoxanthine incorporation assay [9] against the *P. falciparum* strain Cam3.I^R539T^ [12]. The mean IC90 values of DHA, OZ277, OZ439 and OZ609 determined in two independent experiments were 6.0, 4.3, 8.1, 5.1 nM, respectively (or 1.7, 1.7, 3.8 and 2.7 ng/ml), and correlated with the data previously obtained with the sensitive strain NF54 in ≥ 3 independent experiments [12]. Using these experimentally determined IC90 values as a proxy for the MICs, the next step was to consult available concentration–time profiles from clinical trials performed with artesunate (the metabolic precursor of DHA) or synthetic ozonides. DHA exposure profiles from two clinical trials in patients dosed orally with 4 mg/kg artesunate [13, 14] showed that plasma exposures above 700 nM (~ 200 ng/ml) are achievable for about three hours (Additional file 1: Figure S1). Of note, 700 nM is the concentration chosen for the standard RSA [15], and corresponds to ~ 115-fold the MIC (6.0 nM; see above).

Knowing that the maximal in vitro killing rate of artemisinins [16] and ozonides (unpublished data on file with the Medicines for Malaria Venture) is reached at concentrations corresponding to about tenfold the IC50 (equivalent to fivefold the IC90/MIC, or ~ 10 ng/ml), this rather low multiple of the MIC was applied to published concentration–time profiles of DHA. It was found that the fivefold multiple of the IC90/MIC can be maintained for a duration of ~ 6 h in patients (Additional file 1: Figure S1), which corresponds to the 6 h incubation time employed in the standard RSA. An additional concentration, which can be maintained for a duration of ~ 6 h in patients was found to be 25-fold the IC90/MIC, corresponding to ~ 40 ng/ml (Additional file 1: Figure S1). The same MIC multiples were also applied to published concentration–time profiles of the ozonides. From the exposure profile of OZ439 in patients dosed orally with 500 mg [17], it was estimated that 5- and 25-fold the IC90/MIC (~ 20 and ~ 100 ng/ml) were achievable for 48 and 20 h, respectively (Additional file 1: Figure S1). For OZ277, no plasma concentration–time profile from infected patients is published. However, it is known that the activity of the synthetic ozonide OZ277 in clinical studies is in the same order of magnitude as that of the artemisinin derivative artemether in the artemether–lumefantrine combination [8]. Besides, the OZ277 plasma concentrations decline with a similar mean half-life range of 2 to 4 h like DHA [18]. Thus, the same RSA parameters were chosen for OZ277 as for DHA (incubation at 5- and 25-fold the IC90/MIC for a duration of 6 h).

Up to now, OZ609 has not been tested in humans. However, the in vivo half-life of OZ609 is predicted to be in the range of OZ439 (unpublished data on file with the Medicines for Malaria Venture). Hence, for OZ609, the same parameters were chosen as for OZ439 (incubation at 5- and 25-fold the IC90/MIC for 48 and 20 h, respectively).

Having identified clinically relevant compound exposure parameters, the original RSA described by Witkowski et al. [7] was adapted accordingly. Additionally, the drug washout was enhanced to ensure that no residual peroxide was present during the post-treatment period [19]. Moreover, the RSA readout was made less subjective and laborious by replacing the microscopic readout with a semi-automated readout based on [^3^H]hypoxanthine incorporation (details are described in “Methods” section).

**Fig. 1:**
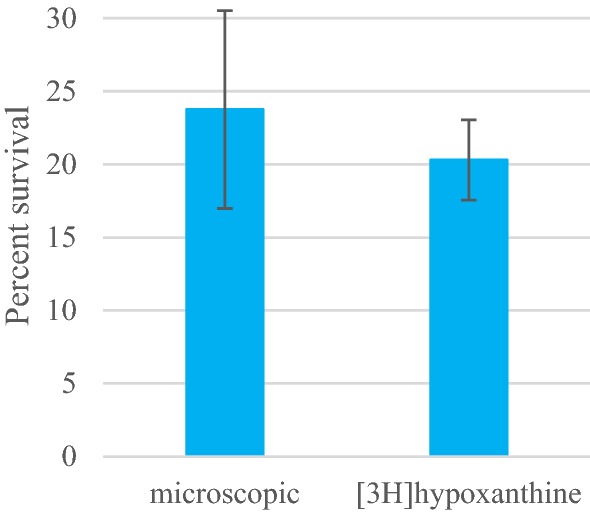
Comparison of the mean survival ± standard deviation after DHA treatment, obtained from the [^3^H]hypoxanthine incorporation method and the conventional microscopic readout with the *Plasmodium falciparum* clinical isolate Cam3.I^R539T^. In all assays, Cam3.I^R539T^ was exposed for 6 h to 700 nM of DHA. For both readouts, the mean values of n = 4 biological replicates are shown

As a first step, the conventional microscopic readout was compared with the [^3^H]hypoxanthine incorporation method by exposing the Cambodian isolate Cam3.I^R539T^ for 6 h to 700 nM of DHA. Both readout methods provided a high survival rate of 20 to 24% (Fig. 1), which is comparable to the observed survival rates of 40%, 33% and 22% published previously [5, 12, 20].

**Fig. 2:**
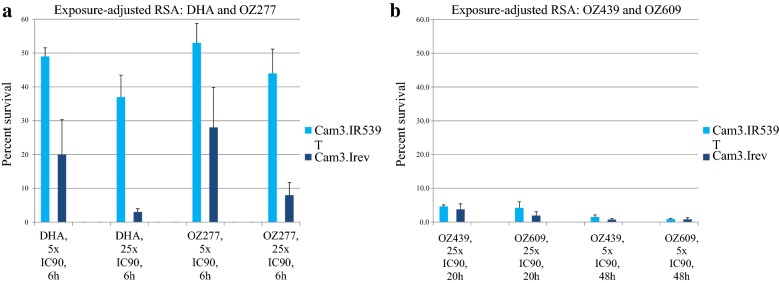
Mean percent survival ± standard deviation of *Plasmodium falciparum* strains Cam3.I^R539T^ and Cam3.I^rev^ after exposure to DHA, OZ277, OZ439 and OZ609 at clinically relevant compound exposure parameters. **a** Parasites were exposed for 6 h to 5- or 25-fold the IC90/MIC of DHA or OZ277 (30 nM or 149 nM DHA; 15 nM or 74 nM OZ277). **b** Parasites were exposed for 20 h to 25-fold or 48 h to 5-fold IC90/MIC of OZ439 or OZ609 (168 nM or 34 nM OZ439; 96 nM or 19 nM OZ609). After drug removal, parasites were first incubated for 22 h with hypoxanthine-free medium, and then for 24 h with medium containing [^3^H]hypoxanthine. After this [^3^H]hypoxanthine incorporation period, the assays were terminated by freezing the plates at − 20 °C. At least three biological replicates were performed in each case

Next, the percent survival of the same parasite isolate was determined after a 6 h exposure to 5- or 25-fold the IC90 (MIC) of DHA or OZ277 and compared with the percent survival of the genetically engineered wildtype strain Cam3.I^rev^. In line with previous observations [12, 20] for both compounds, the clinical isolate Cam3.I^R539T^ strain showed a higher survival than the sensitive Cam3.I^rev^ strain. The difference was most pronounced at 25-fold the IC90/MIC of both compounds (Fig. 2a).

When the two ozonides OZ439 and OZ609 were tested at their clinically relevant concentrations and exposure times (incubated at 5- and 25-fold the IC90/MIC for 48 and 20 h, respectively), both compounds were highly active and showed close-to-equal potency against both strains (Fig. 2b).

## Discussion

This article reports on experiments preformed with the synthetic compounds OZ277, OZ439 and OZ609 against a clinical isolate with a *kelch 13* mutation and shows that, when tested at clinically relevant parameters, OZ439 and OZ609 are active against both the *kelch 13* mutant as well as the wildtype revertant.

At the 48 h time point and with a range of OZ439 concentrations, similar observations were also made by Yang et al. [19]. This is also in line with reports describing that mutations in the *kelch 13* propeller gene did not have an impact on the median parasite clearance half-life rate after treatment with OZ439 [21].

## Conclusion

In conclusion, if the in vitro RSA does indeed predict the potency of compounds against parasites with increased tolerance to artemisinin and its derivatives, then the herein presented data suggest that following drug-pulses of at least 48 h, OZ439 and OZ609 will be highly potent against *kelch 13* mutant isolates such as *P. falciparum* Cam3.I^R539T^. This is consistent with modelling data [22] and recent trial data showing 97.7% efficacy of a 6-day artemisinin-based combination treatment course [23]. Clearly, however, there is a need to constantly monitor the situation in the field by testing ozonides against additional clinical isolates and, importantly, at clinically relevant conditions.

## Supplementary information

**Additional file 1: Figure S1.** Published plasma concentration–time profiles in humans infected with *Plasmodium falciparum* malaria after single-dose, oral treatment. A) Dihydroartemisinin (DHA) profile from reference 13 (Mc Gready et al.); B) Dihydroartemisinin (DHA) profile from reference 14 (Saunders et al.); c) OZ439 profile from reference 17 (McCarthy et al.).

## Data Availability

All data generated or analyzed during this study are included in this published article and its additional information files.

## Abbreviations

RSA: ring-stage survival assay
OZ277: arterolane
OZ439: artefenomel
DHA: dihydroartemisinin
IC50 and IC90: 50 and 90% inhibitory concentrations
MIC: minimal inhibitory concentration
