# Supplementary material for: Anti-malarial ozonides OZ439 and OZ609 tested at clinically relevant compound exposure parameters in a novel ring-stage survival assay
Source: Malar J. 2019 Dec 18;18:427. doi: 10.1186/s12936-019-3056-8 (PMC6918666; doi:10.1186/s12936-019-3056-8)
Supplement: Supplementary file 1 — Additional file 1: Figure S1. Published plasma concentration–time profiles in humans infected with Plasmodium falciparum malaria after single-dose, oral treatment. A) Dihydroartemisinin (DHA) profile from reference 13 (Mc Gready et al.); B) Dihydroartemisinin (DHA) profile from reference 14 (Saunders et al.); c) OZ439 profile from reference 17 (McCarthy et al.). [file 12936_2019_3056_MOESM1_ESM.docx]

**Figure S1: Published plasma concentration–time profiles in humans infected with Plasmodium falciparum malaria after single-dose, oral treatment.** A) Dihydroartemisinin (DHA) profile from reference 13 (Mc Gready et al.); B) Dihydroartemisinin (DHA) profile from reference 14 (Saunders et al.); c) OZ439 profile from reference 17 (McCarthy et al.)
